# Effective Oral Favipiravir (T-705) Therapy Initiated after the Onset of Clinical Disease in a Model of Arenavirus Hemorrhagic Fever

**DOI:** 10.1371/journal.pntd.0001342

**Published:** 2011-10-11

**Authors:** Michelle Mendenhall, Andrew Russell, Donald F. Smee, Jeffery O. Hall, Ramona Skirpstunas, Yousuke Furuta, Brian B. Gowen

**Affiliations:** 1 Department of Animal, Dairy, and Veterinary Sciences, Utah State University, Logan, Utah, United States of America; 2 Department of Agriculture and Food, State of Utah, Logan, Utah, United States of America; 3 Research Laboratories, Toyama Chemical Company, Ltd., Toyama, Japan; Tulane School of Public Health and Tropical Medicine, United States of America

## Abstract

**Background:**

Lassa and Junín viruses are the most prominent members of the *Arenaviridae* family of viruses that cause viral hemorrhagic fever syndromes Lassa fever and Argentine hemorrhagic fever, respectively. At present, ribavirin is the only antiviral drug indicated for use in treatment of these diseases, but because of its limited efficacy in advanced cases of disease and its toxicity, safer and more effective antivirals are needed.

**Methodology/Principal Findings:**

Here, we used a model of acute arenaviral infection in outbred guinea pigs based on challenge with an adapted strain of Pichindé virus (PICV) to further preclinical development of T-705 (Favipiravir), a promising broad-spectrum inhibitor of RNA virus infections. The guinea pig-adapted passage 19 PICV was uniformly lethal with an LD_50_ of ∼5 plaque-forming units and disease was associated with fever, weight loss, thrombocytopenia, coagulation defects, increases in serum aspartate aminotransferase (AST) concentrations, and pantropic viral infection. Favipiravir (300 mg/kg/day, twice daily orally for 14 days) was highly effective, as all animals recovered fully from PICV-induced disease even when therapy was initiated one week after virus challenge when animals were already significantly ill with marked fevers and thrombocytopenia. Antiviral activity and reduced disease severity was evidenced by dramatic reductions in peak serum virus titers and AST concentrations in favipiravir-treated animals. Moreover, a sharp decrease in body temperature was observed shortly after the start of treatment. Oral ribavirin was also evaluated, and although effective, the slower rate of recovery may be a sign of the drug's known toxicity.

**Conclusions/Significance:**

Our findings support further development of favipiravir for the treatment of severe arenaviral infections. The optimization of the experimental favipiravir treatment regimen in the PICV guinea pig model will inform critical future studies in the same species based on challenge with highly pathogenic arenaviruses such as Lassa and Junín.

## Author Summary

Several viruses in the *Arenaviridae* family cause severe life-threatening hemorrhagic fever syndromes, which are considered neglected tropical diseases in endemic areas of Africa and South America. Ribavirin, the only licensed antiviral indicated for use has limited efficacy when treating advanced cases of disease and is associated with toxicity. In the present study, we use a model of acute arenaviral disease in guinea pigs based on infection with an adapted strain of the Pichindé arenavirus (PICV) to further preclinical development of a promising broad-spectrum antiviral drug candidate, favipiravir. Oral favipiravir was highly effective in the treatment of sick animals with marked fevers, as all recovered fully from lethal PICV infection even when therapy was initiated one week after virus challenge. Antiviral activity and reduced disease severity was evidenced by dramatic reductions in serum virus loads and serum aspartate aminotransferase, an enzyme released into the bloodstream following tissue damage and a marker for severe arenaviral infections. Moreover, a sharp decrease in fever was observed shortly after the onset of treatment. Our findings support further development of favipiravir for the treatment of severe arenaviral infections, for which there are presently no safe and effective therapies for treating advanced cases of disease.

## Introduction

A limited number of phylogenetically distinct viruses that belong to the *Arenaviridae*, *Bunyaviridae*, *Filoviridae*, and *Flaviviridae* families can cause a severe hemorrhagic fever syndrome that often results in death. Among the arenaviruses, two Old World (Lassa and Lujo) and several New World (Junín, Machupo, Guanarito, Sabiá, and Chapare) viruses are the etiologic agents of viral hemorrhagic fever in endemic areas of Africa and South America, respectively [1], [2], [3]. Estimates of the number of yearly Lassa virus infections and associated deaths in West Africa range up to 2,000,000 and 10,000, respectively [4]. The highest disease burden in the New World is associated with Junín virus (JUNV) infection in the Pampas agricultural regions of Argentina. Although an effective vaccine has curtailed the number of cases of Argentine hemorrhagic fever (AHF), cases continue to be reported annually [5]. Immune plasma has been used with some success but has been associated with a late neurological syndrome [6]. Ribavirin (1-β-d-ribofuranosyl-1H-1, 2,4-triazole-3carboxamide) is the only licensed antiviral with reported activity against Lassa virus (LASV), JUNV, and Machupo virus (MACV) [7], [8], [9], and could be used off-label in the event of an emergency [10]. Although the adverse effects in humans treated with ribavirin are generally considered to be mild and reversible with termination of treatment [11], [12], [13], [14], teratogenicity and embryotoxicity are of concern [15], [16]. In addition, intravenous ribavirin is not widely available and is often very expensive [17].

Favipiravir (T-705; 6-flouro-3-hydroxy-2-pyrazinecarboxamide) is a pyrazine derivative presently being developed clinically for the treatment of influenza virus infections. Orally administered favipiravir has shown efficacy in experimental mouse and hamster models of arenavirus, phlebovirus, flavivirus, and influenza virus infections [18], [19], [20], [21], [22], [23]. We were able to demonstrate a limited protective antiviral effect when treating advanced Pichindé virus (PICV) infection in hamsters [24]. In Vero cell culture experiments, we have demonstrated micromolar range activity of favipiravir against the JUNV vaccine strain, Candid 1 [19]. We have now confirmed this activity with a pathogenic strain of JUNV, as well as isolates of other South American hemorrhagic fever viruses [25].

Although several groups have recently reported on the development of mouse models of LASV and JUNV infection, these systems are based on challenge of immunocompromised animals [26], [27]. Guinea pig infection models have been described for LASV, JUNV and Guanarito virus (GTOV) [28], [29], [30], [31], and are the best-suited small animal models to further investigate the activity of favipiravir. Due to the maximum containment requirement and high costs associated with conducting studies with highly pathogenic arenaviruses, the aim of the present work was to evaluate favipiravir in the guinea pig PICV infection model [32], [33], as a means to optimize treatment conditions to assist in the planning of future studies in biosafety level 4 (BSL-4) containment. Our PICV stock derived from a single additional passage of a previously described guinea pig-adapted virus [33] was uniformly lethal in outbred guinea pigs, and we characterized the natural history of disease to establish the model in our laboratory for use in the evaluation of favipiravir.

## Methods

### Ethics statement

All animal procedures complied with USDA guidelines and were conducted at the AAALAC-accredited Laboratory Animal Research Center at Utah State University under protocol 1393, approved by the Utah State University Institutional Animal Care and Use Committee.

### Animals

Outbred male Hartley strain guinea pigs weighing ∼300–350 g were obtained from Charles River (Wilmington, MA). Animals were sorted prior to the start of all experiments so that the average group weight was similar across all groups. For all experiments, IPTT-300 electronic transponders were subcutaneously implanted for identification and temperature measurement in conjunction with the DAS 6002 scanner (BMDS, Seaford, DE).

### Virus

Guinea pig-adapted PICV, passage 18 (p18), was provided by Dr. Robert Tesh (World Reference Center for Emerging Viruses and Arboviruses, University of Texas Medical Branch, Galveston, TX). The p18 strain was derived from 2 additional passages of a p16 guinea pig spleen suspension of the CoAn 4763 Munchique strain obtained from the U.S. Army Medical Research Institute of Infectious Diseases (USAMRIID). A p19 spleen homogenate was prepared from a single ill p18-infected guinea pig euthanized on day 12 post-infection. The p19 stock (∼4.8×10^6^ plaque-forming units (PFU)/ml) was used for all challenge studies. Sequencing of viral RNA isolated from the p19 stock was performed by SeqWright DNA Technologies Services (Houston, TX) using standard fluorescent dye-terminator DNA sequencing chemistry following RT-PCR amplification. GenBank accession numbers for the p19 S and L segments are JN378747 and JN7378748, respectively.

### Test compounds

Favipiravir (T-705) was provided by the Toyama Chemical Company, Ltd. (Tokyo, Japan). Ribavirin was supplied by ICN Pharmaceuticals, Inc. (Costa Mesa, CA). Both were suspended in GERBER NatureSelect 1st FOODS carrot food (ingredients: carrots and water) for oral administration.

Favipiravir toxicity was assessed in guinea pigs following twice-daily treatments for 10 days. Groups of five guinea pigs each were dosed orally with 500, 250, 100 and 0 (placebo) mg/kg/day of favipiravir. Treatments were administered using 1 ml tuberculin syringes by placement of the doses in carrot food vehicle in the back of the oral cavity. During the 10-day dosing period and for seven days following, guinea pigs were monitored closely for signs of toxicosis and weights and temperatures were recorded daily. Seven days after the final dose was administered, animals were euthanized by CO_2_ asphyxiation, whole blood and serum were collected for hematology and blood chemistry analyses, and necropsies and pathological examination were performed at the Ross A. Smart Veterinary Diagnostic Laboratory (Logan, UT).

### PICV titration and natural history studies

Virus titrations were performed to determine 50% and 90% lethal doses (LD_50_ and LD_90_) of the p19 PICV stock. Groups of three to four guinea pigs each were challenged by bilateral intraperitoneal (i.p.) injections with log_10_ PICV quantities ranging from 0.05 to 50,000 PFU prepared in minimal essential medium (MEM). Body weight and temperature, and morbidity and mortality were monitored for 28 days following infection. Clinical signs of illness were weight loss, pyrexia, ruffling of fur, and lethargy. In this and all other experiments, animals were considered moribund and euthanized when they lost 20% of their starting body weight or their body temperature dropped to 36°C or less. For survival analysis, animals were counted as dead the day after euthanasia. LD_50_ and LD_90_ values were determined by regression analysis.

Based on the titration data, guinea pigs were infected with 500 PFU of PICV for the natural history study and the challenge efficacy experiments. For the natural history study, PICV-challenged guinea pigs were sacrificed daily (n = 3/day), with the exception of day 10 and 11 of infection, on which 4 and 2 guinea pigs were euthanized, respectively, due to one of the day 11 animals having reached the 20% weight loss euthanasia criteria on day 10. Whole blood (in both citrate- and EDTA-coated tubes; Sarstedt Inc., Newton, NC), serum, livers, lungs, kidneys, spleens, and brains were harvested. Whole spleens were weighed prior to sectioning. Sections from each tissue were preserved in 10% formalin and sent to the Ross A. Smart Veterinary Diagnostic Laboratory (Logan, UT) for histologic analysis. The other sections were stored at −80°C and virus titers determined as described below. Whole blood was analyzed for coagulation and hematologic parameters, and serum was analyzed for viremia and comprehensive blood chemistry as described below.

### Favipiravir efficacy studies

Two independent studies were performed to investigate the efficacy of favipiravir in guinea pigs challenged with PICV. In the first study, groups of 8 guinea pigs each were treated twice daily with 100 and 30 mg/kg/day of favipiravir on days 4–7 of infection. Due to continued deterioration of the animals despite favipiravir treatment, the doses were increased to 300 and 90 mg/kg/day, respectively, for the remainder of the treatment schedule (days 8–17). For comparison, 8 guinea pigs each were treated twice daily with 50 mg/kg/day of ribavirin or carrot food placebo on days 4–17 after challenge. Body weight and temperature, and morbidity and mortality were monitored for 29 days post-challenge. Serum was collected by saphenous vein puncture from all animals on day 11 of infection, with the exception of two animals in the placebo group that had to be put down on day 10. Serum was analyzed for viral burden, aspartate aminotransferase (AST) and alanine aminotransferase (ALT) as described below. Serum was also collected from surviving animals at the conclusion of the experiment (day 29) for virus titer analysis.

In the second study, groups of 8 guinea pigs each were treated orally with 300 or 150 mg/kg/day favipiravir, or vehicle placebo, divided into two daily doses for 14 days beginning on day 7 after infection. In an attempt reduce the high viral loads encountered at the start of treatment, the 150-mg/kg/day favipiravir group received a loading dose of 300 mg/kg on the first day, with a shift to the lower maintenance dose thereafter. This strategy is commonly used in the clinic and has been employed for the treatment of cases of Lassa fever and AHF [17], [34]. A group of 7 guinea pigs received 50 mg/kg/day of ribavirin, with treatment also starting on day 7 and given twice daily for 14 days. Guinea pigs were monitored for signs of illness and weights and temperatures were recorded as previously described. Serum was collected on day 10 from all animals by saphenous vein puncture and analyzed for viremia and AST concentration. Sera, spleens, livers, lungs, kidneys, and brains were collected from surviving animals at the end of the study (day 36) for virus titer analysis.

### Serum and tissue virus titers

Virus titers were determined using an infectious cell culture assay as previously described [19]. Briefly, tissues were homogenized 1∶10 w/v in MEM. Serum and homogenized tissue samples were serially log_10_ diluted and plated in triplicate wells on Vero cell monolayers (American Type Culture Collection, Manassas, VA) in 96-well microtiter plates. Plates were incubated for 7 days and viral cytopathic effect (CPE) was determined for calculation of 50% endpoints by the Reed-Muench method [35]. Assay detection range was 1.8 to 8.5 log_10_ 50% cell culture infectious dose (CCID_50_)/ml of serum or 0.1 g of tissue.

### Coagulation, hematology, and blood chemistry

Whole blood in citrate-coated tubes was analyzed for prothrombin time (PT) and activated partial thromboplastin time (aPTT) using the VetScan VSpro and PT/aPTT cartridges (Abaxis Inc., Union City, CA). Due to a technical problem, PT clot times were not detected in two of three animals on day 1.

For the characterization and efficacy studies, whole blood in EDTA-coated tubes was analyzed for hematology using the VetScan HMT (Abaxis Inc.). Hematology factors included white blood cells (WBC), lymphocytes (Lym), monocytes (Mon), granulocytes (Gra), red blood cells (RBC), mean corpuscular volume (MCV), hematocrit (Hct), mean corpuscular hemoglobin (MCH), mean corpuscular hemoglobin concentration (MCHC), red cell distribution width (RDW), hemoglobin (Hb), platelets (PLT), mean platelet volume (MPV), plateletcrit (PCT), and platelet distribution width (PDW). For the favipiravir toxicity study, hematology was performed using a HEMAVET HV 950 (Drew Scientific, Dallas, TX) and parameters evaluated were equivalent to those above, except WBC were further broken down to include neutrophils (Neu), eosinophils (Eos) and basophils (Bas).

Serum was analyzed for comprehensive blood chemistry or individual analytes using the DRI-CHEM 4000 chemistry analyzer (Heska, Loveland, CA). Blood analytes included AST, amylase (AMY), alkaline phosphatase (ALP), ALT, blood urea nitrogen (BUN), calcium (Ca), creatinine (CRE), gamma-glutamyl transferase (GGT), glucose (GLU), total protein (TP), total bilirubin (TBIL), albumin (ALB), cholesterol (CHO), and inorganic phosphate (IP). For the favipiravir toxicity study, blood chemistry analysis was performed using the VetScan VS2 and comprehensive diagnostic profile rotors (Abaxis Inc.). Similar analytes were profiled with the exception of CHO and the addition of phosphate (PHOS), sodium (Na^+^), potassium (K^+^), and globulin (GLOB).

### Pharmacokinetic analysis of orally administered favipiravir in guinea pigs by HPLC

Three guinea pigs were treated by placement of a 50 mg/kg dose of favipiravir, suspended in carrot food vehicle, towards the back of the oral cavity with a 1 ml syringe. Plasma was collected from each animal at 15 min, 30 min, 1 h, 2 h, and 4 h by saphenous vein puncture. Each sample was mixed with equal volume of 1∶1 methanol∶acetonitrile for deproteinization. Samples were centrifuged (10,000× *g*) for 10 min and supernatants transferred to new tubes for evaporation. The contents were then resuspended in HPLC buffer for analysis as previously described [24]. Favipiravir plasma concentrations were extrapolated using a standard curve from samples containing known amounts of favipiravir. Area under the curve (AUC) analysis and half-life (t_1/2_) estimation were performed using Prism (GraphPad Software, La Jolla, CA).

### Statistical analysis

The Mantel-Cox log-rank test was performed to analyze the survival data. Hematology, blood chemistry, virus titer, coagulation, and spleen weight data were analyzed using one-way analysis of variance (ANOVA) followed by Bonferroni multiple comparison test. Correlation of PLT count with other platelet parameters (PCT, MPV and PDW) were performed according to the Pearson rank correlation method. All statistical evaluations were done using Prism (GraphPad Software).

## Results

### Characterization of lethal PICV infection in guinea pigs

**Figure 1:**
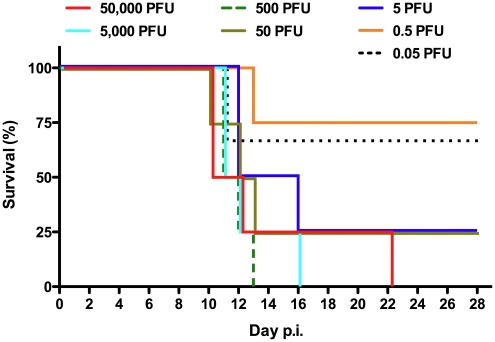
Titration of PICV in outbred guinea pigs. Hartley guinea pigs were challenged i.p. with 50,000, 5000, 500, 50, 5, 0.5 (n = 4/group), or 0.05 PFU (n = 3) p19 PICV and survival plotted over a 28-day post-infection (p.i.) period. The data are combined from two separate experiments.

Because of the reported variability in lethality caused by PICV infection of outbred guinea pigs [32], [36], [37], [38], [39], we sought to establish a uniformly lethal model that would facilitate the evaluation of favipiravir. We prepared a virus stock from a single passage of the p18 guinea pig-adapted strain in Hartley guinea pigs. The p19 PICV was derived from the spleen of clinically ill guinea pig with an advanced infection on day 12 post-challenge. The p19 stock was found to be highly virulent causing severe disease in guinea pigs with 100% mortality at challenge doses ≥500 PFU (Figure 1). The LD_50_ of the p19 stock was ∼5 PFU with an LD_90_ of ∼200 PFU. Complete sequence analysis of the L and S segments from the p19 virus stock revealed only a single substitution in the consensus sequence compared to the previously reported p18 sequences [39]. The substitution was present in the L segment and was heterogeneous matching either the p18 or p2 sequences.

**Figure 2:**
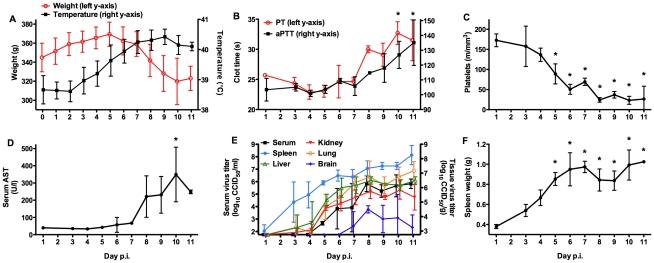
Natural history of disease caused by PICV infection in guinea pigs. Animals were challenged with 500 PFU of p19 PICV, (A) daily weights and temperatures were recorded, and groups of 3 were sacrificed on the indicated day p.i. for collection of blood and tissues for analysis of (B) PT and aPTT clot times, (C) PLT counts, (D) serum AST levels, (E) infectious serum and tissue virus titers, and (F) splenomegaly during the course of infection. Due to a technical problem, PT clot times were not detected in two of three animals on day 1. *P<0.05 compared to day 1 p.i. (day 3 p.i. for PT).

We next investigated the natural history of disease in guinea pigs challenged with 500 PFU of the p19 virus. PICV-induced disease in guinea pigs was marked by elevated temperatures beginning on day 3 after infection, with weight loss and anorexia becoming evident by day 6 (Figure 2A). Clot times for extrinsic (PT) and intrinsic (aPTT) coagulation pathways were increased as the infection progressed past day 7 (Figure 2B). Hematological analysis revealed additional alterations affecting the coagulation system. Marked thrombocytopenia was observed starting on day 5 post-infection with dramatic decreases in platelet counts (PLT; Figure 2C, Table S1) and plateletcrit (PCT; Table S1); the latter measure being directly related to the total number of platelets. Platelet distribution width (PDW), a marker for platelet activation [40], concomitantly decreased and became increasingly variable as the infection progressed and, due to low PLT, could not be measured past day 7 (Table S1). On the other hand, mean platelet volume (MPV), considered an indicator of platelet function [41], did not significantly change over the course of the course of the study and did not correlate with PLT (Table S1). PLT concentration had a strong correlation with both PCT (*r* = 1.0; *P* = 0.0001) and PDW (*r* = 0.83; *P* = 0.0015). The only other notable hematologic findings observed were spikes in total WBC and granulocytes (Gra) on day 7 of infection; however, both parameters returned to the normal range the following day (Table S1).

In addition to the depleted PLT and PCT levels, and the prolonged coagulation times observed during the course of acute PICV infection, we found blood in the stools of several ill animals with advanced clinical disease signs (ruffling of fur and anorexia). Evidence of hemorrhaging within individual tissues was not observed upon pathological examination, suggesting that internal bleeding into tissues was not contributing substantially to the demise of the animals. Severe hemorrhagic manifestations are not often seen in human arenaviral hemorrhagic fever cases [42], [43].

Comprehensive blood chemistry analysis revealed a dramatic increase in serum AST concentration starting on day 8 of PICV infection (Figure 2D). In contrast, serum ALB decreased gradually through the course of the infection (Table S2), which may reflect alterations in vascular permeability or nutritional status. No other significant changes in the blood chemistry parameters evaluated were observed (Table S2).

Viremia and tissue virus titers were also assessed on a daily basis. As shown in Figure 2E, PICV replication was observed in all tissues examined. Onset of viremia occurred on day 5 post-challenge and persisted through day 11. The spleen supported vigorous replication as PICV could be detected as early as day 1, and having the greatest viral loads throughout the acute infection period, with titers of >10^8^ CCID_50_/g. The liver, lungs, and kidneys also had substantial viral burdens that crested on day 8 and persisted through the end of the study. Infectious virus was detectable in the brain of several animals starting on day 7, but the low titers may be attributable to blood-borne virus. This trend was similarly observed in LASV-infected and PICV (p8 strain)-infected strain 13 guinea pigs [28], [32].

Consistent with the high viral loads measured in spleen tissue homogenates, the spleens of PICV-infected guinea pigs were grossly enlarged and weighed significantly more on days 5–11 of infection compared to day 1 (Figure 2F). As described in previous studies using lower passage strains of adapted PICV and inbred strain 13 guinea pigs [32], [44], we also found the liver and spleen to be most affected histologically by p19 PICV infection (not shown), although the degree of damage in these tissues was minor and less than would have been expected based on viral titers (Figure 2E). Few to moderate numbers of necrotic cells were observed in the interstitium and periarteriolar sheaths of the spleen and acute multifocal hepatic necrosis was observed as early as day 6 and necrotic areas were more prominent in later days of infection (not shown). Several guinea pigs exhibited hepatic lipidosis on days 10 and 11; however, this change is most likely due to the mobilization of fat for energy as the sick animals greatly reduce food consumption. All other tissues appeared normal upon histologic examination. Our observations are consistent with those noted in human Lassa fever, in which histopathologic findings are generally not severe enough to account for death [45].

### Favipiravir therapy of guinea pigs challenged with a lethal dose of PICV

Having established a uniformly lethal guinea pig PICV infection model and characterized the timing of disease progression, the second objective was to evaluate the efficacy of oral favipiravir using this model. Because of a small palatal ostium, oral gavage of guinea pigs is very difficult and generally contraindicated. Thus, we devised a method to treat the animals by suspending test drug in a carrot food vehicle for administration as described in detail in the methods section. Using this method of drug delivery, we were able to confirm gastric absorption of favipiravir (AUC = 44 µg/ml h; t_1/2_ = 1.42 h) with peak plasma levels in the range of 40 µg/ml (256 µM) within 15–30 min of treatment with a 50-mg/kg dose (Figure S1).

**Figure 3:**
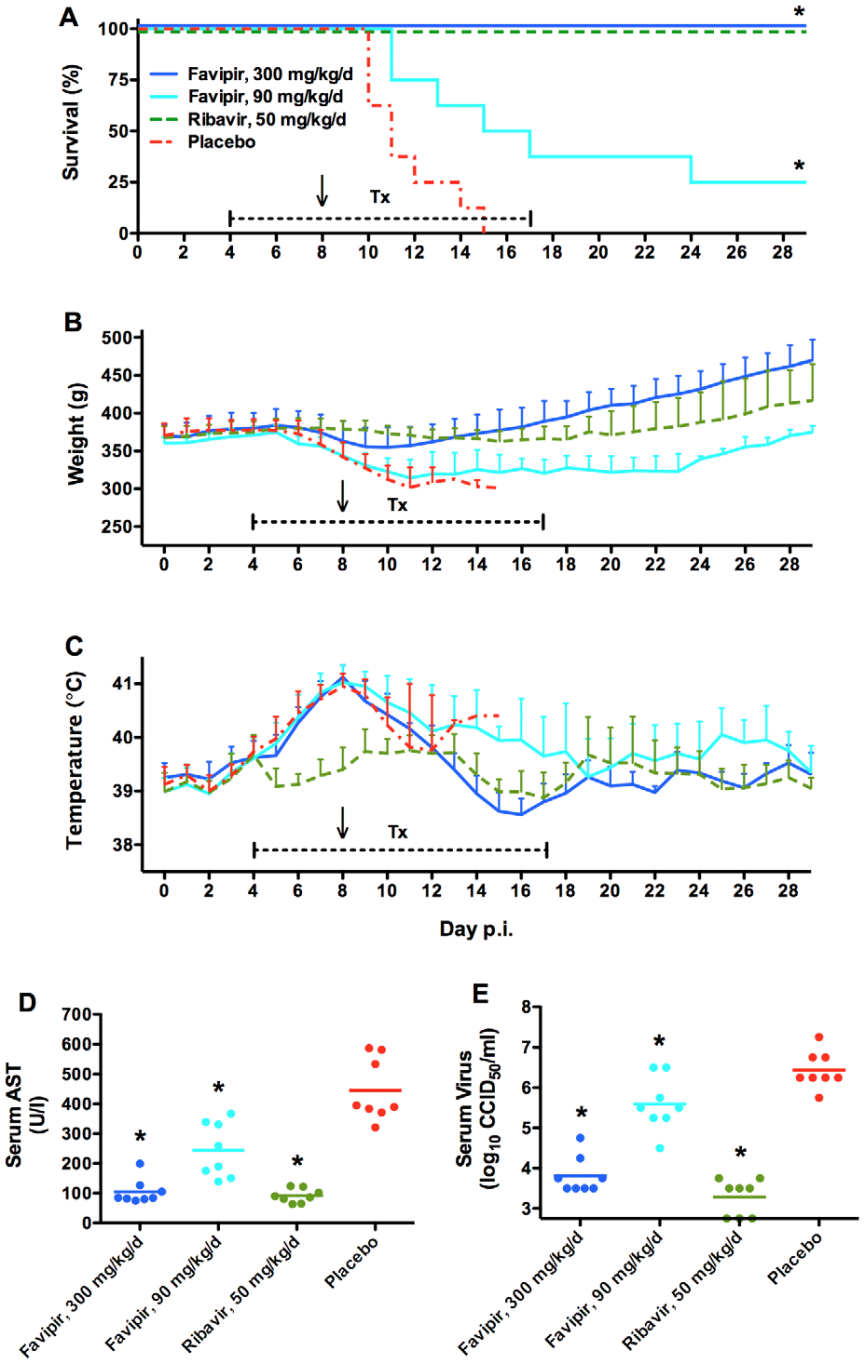
Treatment of lethal PICV infection with favipiravir starting 4 days after challenge. Guinea pigs (n = 8/group) challenged with 500 PFU of p19 PICV were treated twice daily for 14 days with favipiravir, ribavirin, or placebo beginning on day 4 of infection (capped hashed line). Animals receiving favipiravir were initially fed 100 and 30 mg/kg/d (days 4 to 7) before increasing the doses to 300 and 90 mg/kg/d, respectively, starting on day 8 (indicated by arrow). (A) Survival, (B) body weights, and (C) temperatures were monitored for 29 days. Serum was collected on day 11 for analysis of (D) AST and (E) viremia, with the exception of two moribund animals from the placebo group that had to be sacrificed on day 10. *P<0.05 compared to placebo-treated animals.

To reach favipiravir concentrations well above the reported 50% effective concentration (EC_50_) of ∼17 µM for arenavirus inhibition in cell culture [19], [46], we treated PICV-infected guinea pigs with 100 or 30 mg/kg/day. This dose range has also been previously shown to be effective in treating PICV infection in hamsters by oral gavage [24]. Favipiravir, placebo, and ribavirin (positive control) treatments were initiated 4 days after challenge and dosed twice daily for 14 days (Figure 3A). Because we did not observe any signs of toxicity following 10 days of treatment with a favipiravir dose of 500 mg/kg/day (Table S3), we extended the duration of treatment to facilitate complete clearance of the virus. Unexpectedly, despite 4 days of therapy, guinea pigs treated with favipiravir began to lose weight, became lethargic, and developed high fevers similar to animals treated with placebo (Figure 3B, C). In contrast, guinea pigs treated with ribavirin (50 mg/kg/day) did not develop fever or show signs of illness. Consequently, we decided to triple the dose of favipiravir for the remaining 10 days of therapy starting on the morning of day 8 post-infection, as no clinical signs of adverse effects, histopathology (not shown), or changes in laboratory values were observed in toxicity studies when guinea pigs were treated with up to 500 mg/kg/day (Table S3).

The increase in the favipiravir dose resulted in a rapid reduction in fever in the 300-mg/kg/day group (Figure 3C), with all animals recovering completely (Figure 3A), as reflected by robust weight gain at a rate greater than ribavirin (Figure 3B). The 90-mg/kg/day dose of favipiravir provided a reduced, yet significant degree of protection. Notably, the reduction in fever seen starting on day 9 for both the placebo and low-dose favipiravir groups is principally due to decreasing temperatures in animals as they become moribund (Figure 3C). Guinea pigs receiving ribavirin therapy all survived the challenge, but recovered more slowly as demonstrated by the shallower weight gain trend relative to the high-dose favipiravir group. Serum AST and virus titers measured on day 11 were significantly lower in all drug-treated groups compared to the placebo, with a clear dose response evident with the favipiravir-treated animals (Figure 3D, E). All surviving guinea pigs had undetectable virus in the serum at the conclusion of the experiment (not shown).

**Figure 4:**
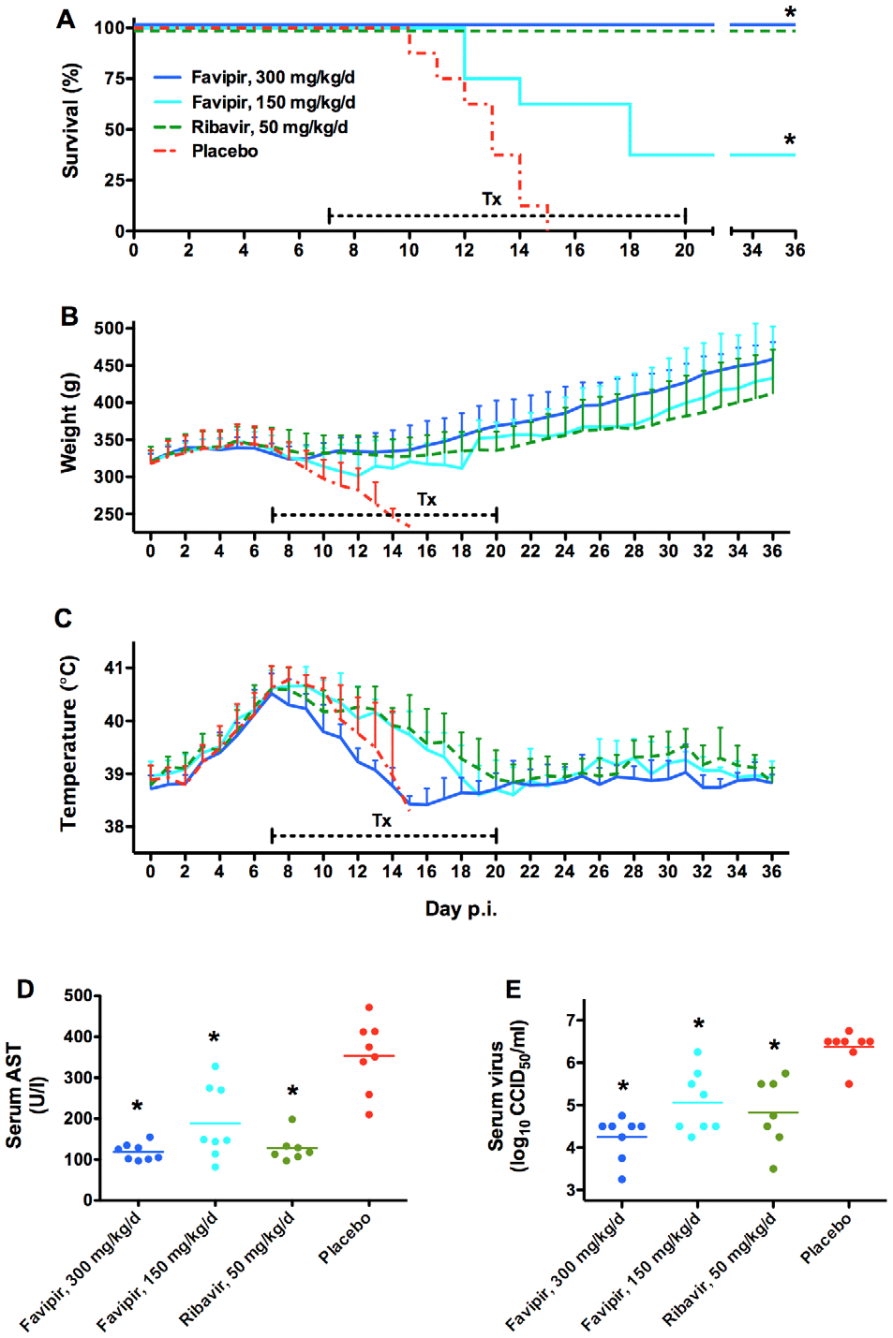
Favipiravir treatment of advanced PICV infection in guinea pigs. Guinea pigs (n = 7–8/group) challenged with 500 PFU of p19 PICV were treated with the indicated dosages of favipiravir, ribavirin, or placebo beginning on day 7 of infection. The 150-mg/kg/d group received a loading dose of 300 mg/kg/d on the first day of treatment. Drugs were administered twice daily for 14 days (capped hashed line) and (A) survival, (B) body weights, and (C) temperatures were monitored for 36 days. Serum was collected on day 10 for analysis of (D) AST and (E) viremia. *P<0.05 compared to placebo-treated animals.

A second efficacy study was conducted wherein treatment was initiated one week after virus challenge, to assess the ability of favipiravir to treat more advanced PICV infection and disease in guinea pigs exhibiting clear clinical signs of illness, including anorexia and sustained fever. Similar to the first experiment, we were able to successfully treat lethal PICV challenge with a 300 mg/kg/day favipiravir regimen (Figure 4A), even when delaying treatment until a time when animals presented with considerable viral loads, splenomegaly, and were thrombocytopenic, febrile, and losing weight (Figure 2A, C, E, F). The intermediate dose of 150 mg/kg/day of favipiravir, which included a 300-mg/kg loading dose on the first day of treatment, also provided a significant level of protection (Figure 4A). All animals treated with ribavirin at 50 mg/kg/day survived the challenge.

Most guinea pigs began to lose weight beginning on day 6 after PICV challenge (Figure 4B). The high-dose favipiravir group decreased in body weight until day 9, at which point the animals began to steadily recover throughout the remainder of the experiment. Guinea pigs in the intermediate-dose favipiravir treatment group that succumbed to illness steadily decreased in body weight, while surviving counterparts began to recover as early as day 11. Guinea pigs in the ribavirin treatment group maintained fairly steady weights through day 21, when they began to gradually gain weight through the rest of the observation period. In both studies, slower rate of weight gain compared to the high-dose favipiravir group was observed. Animals in the placebo group sharply decreased in body weight prior to succumbing to the infection.

Most guinea pigs presented with elevated temperatures of >40°C by day 6 of PICV infection (Figure 4C). Similar to the initial efficacy study, fever was almost immediately reduced following the onset of therapy with 300 mg/kg/day of favipiravir. As before, it is important to note that prior to succumbing to the infection, animals in the intermediate-dose favipiravir and placebo groups dropped in temperature as they approached the terminal stage of the disease.

Serum AST, reflective of the extent of tissue damage, was significantly lower in all drug-treated groups compared to the placebo when measured on day 10 post PICV challenge (Figure 4D). Serum virus titers were also significantly lower in all treatment groups, with average titers of 6.4 log_10_ CCID_50_/ml in the placebo, and 4.3, 5.1, and 4.8 log_10_ CCID_50_/ml in the high- and intermediate-dose favipiravir, and ribavirin groups, respectively (Figure 4E). The treated guinea pigs that survived the 36 day observation period were all found to be devoid of systemic and tissue virus titers (not shown).

## Discussion

Because there are presently no other small animal model options based on challenge of immune competent animals with highly pathogenic viral hemorrhagic fever-causing arenaviruses, future studies evaluating favipiravir as an antiviral therapy for the treatment of severe arenaviral infections will likely first be done in guinea pig JUNV, GTOV, or LASV infection models that require BSL-4 maximum biocontainment facilities [47]. To this end, we sought to establish a guinea pig PICV infection model similar to those previously described [32], [33]. PICV infection in guinea pigs has proven to be useful for the study of acute arenaviral disease [37], [38], [39], [44] and for preclinical efficacy evaluations [48], [49], as the virus can be handled safely in BSL-2 containment. There have been mixed reports on the lethality of guinea pig-adapted PICV in the readily available Hartley outbred guinea pig strain [32], [36], [37], [38], [39]. In addition to other factors, the variation in the stringency of the criteria used to define the terminal endpoints likely contributed to the reported variability. We found that our PICV stock prepared from the spleen of a clinically ill Hartley guinea pig sacrificed on day 12 of infection was uniformly lethal when inoculated at i.p. at doses of 500 PFU or more, but the genetic make-up of the virus did not vary substantially from previously reported sequences. Because most of the previous studies with PICV in guinea pigs that investigated pathogenesis, pathophysiology, virology, and clinical chemistry have used inbred animals that varied in age, gender, and/or infectious dose of PICV, we first characterized the p19 PICV infection in male 350 g outbred guinea pigs. The results provided a detailed picture into the evolution of the clinical disease and pathophysiology specific to the present model, facilitating the evaluation of favipiravir with the goal of demonstrating anti-arenavirus activity in guinea pigs and optimizing the dosing method, level, frequency, and duration of treatment to inform future BSL-4 studies.

With an understanding of the natural history of disease in our guinea pig p19 PICV infection model, we assessed the anti-arenavirus activity of orally administered favipiravir. Although previously we were able to show limited efficacy with favipiravir in treating advanced PICV disease in a hamster model [24], here we were able to demonstrate complete protection from lethal disease in guinea pigs when treatment was initiated well after the onset of fever and the beginning of weight loss. Notably, PICV-infected hamsters do not develop fever, and weight loss is not apparent until the day prior to death [50]. To this end, the insidious progression of the human disease is better reflected in guinea pigs, including the development of fever, a hallmark of the clinical arenaviral hemorrhagic fever diseases. Importantly, favipiravir significantly reduced viremia and systemic AST concentrations, which are prognostic indicators for severe disease and lethality in Lassa fever patients [51]. The efficacy studies conducted provide the foundation for more advanced evaluations with favipiravir employing guinea pig infection models based on challenge with authentic arenaviral hemorrhagic fever viruses, such as the JUNV-guinea pig model actively being used to study AHF [30].

Although we were able to effectively treat PICV-infected guinea pigs with favipiravir, the dosage required was higher than expected based on previous studies in hamsters [19], [24]. A dose of 300 mg/kg/day was needed to achieve 100% survival in guinea pigs challenged with PICV, whereas a 100-mg/kg/day therapeutic regimen afforded the same level of protection when initiated as late as day 5 following infection in hamsters [24]. To put these findings into perspective, the dosage presently being used for clinical evaluation of favipiravir for influenza treatment in humans is 40 mg/kg/day on the first day, with a reduction to 27 mg/kg/day for an additional 4 days. Nevertheless, the equivalent 300-mg/kg/day guinea pig dosage based on body surface area translation [52] would be 65 mg/kg/day. For hamsters, the 100-mg/kg/day dosage would be 14 mg/kg/day.

The higher dose requirement of favipiravir in the guinea pig model is not likely due to differences in the virus stocks used, as our analysis of the An 4763 strain used to infect hamsters and the p19 guinea-pig adapted strain used in the present study were equally sensitive to the inhibitory effects of favipiravir in cell culture (M. Mendenhall, unpublished data). The evidence to date suggests that favipiravir acts as a purine nucleoside analog targeting the viral RNA-dependent RNA polymerase [25], [53]. We hypothesize that the difference in effective dosage of favipiravir is most likely due to a less efficient conversion of the parent compound, T-705, to its active triphosphate form (T-705RTP) in guinea pigs, and/or a more rapid systemic elimination. However, we cannot rule out the possibility of better absorption and more favorable biodistribution of favipiravir in 0.4% carboxymethyl cellulose vehicle when given by oral gavage, as previously described for hamsters [19], [24]. Notwithstanding, our present findings, coupled with having recently demonstrated favipiravir activity in cell culture against JUNV, GTOV, and MACV [25], have us positioned to investigate activity against arenaviruses that are the etiologic agents of Argentine and Venezuelan hemorrhagic fevers in humans, in existing guinea pig infection models [30], [31].

## Supporting Information

Figure S1 **PK analysis of favipiravir in guinea pigs dosed by oral cavity placement.** Favipiravir (50 mg/kg) was prepared in carrot food vehicle and administered by placement of the dose towards the back of the palate with a tuberculin syringe. Plasma was collected at 0.25, 0.5, 1, 2 or 4 h after treatment from 3 guinea pigs treated on 2 separate days. Samples were processed and analyzed by HPLC for separation and measurement of favipiravir as described in the methods section.
(TIFF)

Table S1 **Hematological values during the course of PICV infection in guinea pigs^a^.** ^a^ Groups of guinea pigs (n = 3) were sacrificed daily through the course of PICV infection and whole blood was collected for hematologic analysis. ^b^ The day-10 group (n = 4) included a moribund guinea pig from the day-11 group. ^c^ The day-11 group consisted of 2 guinea pigs. ^d^ Total WBC and Gra were significantly increased on day 7 compared to day 1 (*P*<0.05). ^e^ PLT and PCT were significantly lower on days 5–10 and 6–11, respectively, compared to day 1 (*P*<0.05). ^f^ PDW could not be accurately calculated due to depleted platelet counts on days 8–11. WBC, white blood cells; Lym, lymphocytes; Mon, monocytes; Gra, granulocytes; RBC, red blood cells; MCV, mean corpuscular volume; HCT, hematocrit; MCH, mean corpuscular hemoglobin; MCHC, mean corpuscular hemoglogin concentration; RDW, red cell distribution width; Hb, hemoglobin; PLT, platelets; MPV, mean platelet volume; PCT, plateletcrit; and PDW, platelet distribution width.
(DOC)

Table S2 **Blood chemistry profile during PICV infection in guinea pigs^a^.** ^a^ Groups of guinea pigs (n = 3) were sacrificed daily through the course of PICV infection and whole blood was collected for blood chemistry analysis. ^b^ The day-10 group (n = 4) included a moribund guinea pig from the day-11 group. ^c^ The day-11 group consisted of 2 guinea pigs. ^d^ Serum AST was significantly higher on day 10 compared to day 1 (*P*<0.05). ^e^ Serum ALB was significantly lower on days 9 and 10 compared to day 1 (*P*<0.05). ^f^ Values were below the limit of detection (50 mg/dL) in all three guinea pigs. AST, aspartate aminotransferase; AMY, amylase; ALP, alkaline phosphatase; ALT, alanine aminotransferase; BUN, blood urea nitrogen; ALB, albumin; TBIL, total bilirubin; Ca, calcium; CRE, creatinine; GGT, gamma-glutamyl transferase; GLU, glucose; TP, total protein; TBIL, total bilirubin;. ALB, Albumin; CHO, cholesterol; IP, inorganic phosphate.
(DOC)

Table S3 **Hematology and blood chemistry analysis of guinea pigs treated for 10 days with favipiravir^a^.** ^a^ Guinea pigs (n = 5/group) treated orally twice daily for ten days with the indicated doses of favipiravir and sacrificed 7 days after the final dose was administered. Whole blood and sera were analyzed for hematology and blood chemistry. WBC, white blood cells; Neu, neutrophils; Lym, lymphocytes; Mon, monocytes; Eos, eosinophils; Bas, basophils; RBC, red blood cells; Hb, hemoglobin; HCT, hematocrit; MCV, mean corpuscular volume; MCH, mean corpuscular hemoglobin; MCHC, mean corpuscular hemoglogin concentration; RDW, red cell distribution width; PLT, platelets; MPV, mean platelet volume; ALB, albumin; ALP, alkaline phosphatase; ALT, alanine aminotransferase; AMY, amylase; TBIL, total bilirubin; BUN, blood urea nitrogen; Ca, calcium, PHOS, phosphate; CRE, creatinine; GLU, glucose; Na^+^, sodium; K^+^, potassium; TP, total protein; GLOB, globulin.
(DOC)
