# Supplementary figures and images for: Effective Oral Favipiravir (T-705) Therapy Initiated after the Onset of Clinical Disease in a Model of Arenavirus Hemorrhagic Fever
Source: PLoS Negl Trop Dis. 2011 Oct 11;5(10):e1342. doi: 10.1371/journal.pntd.0001342 (PMC3191123; doi:10.1371/journal.pntd.0001342)

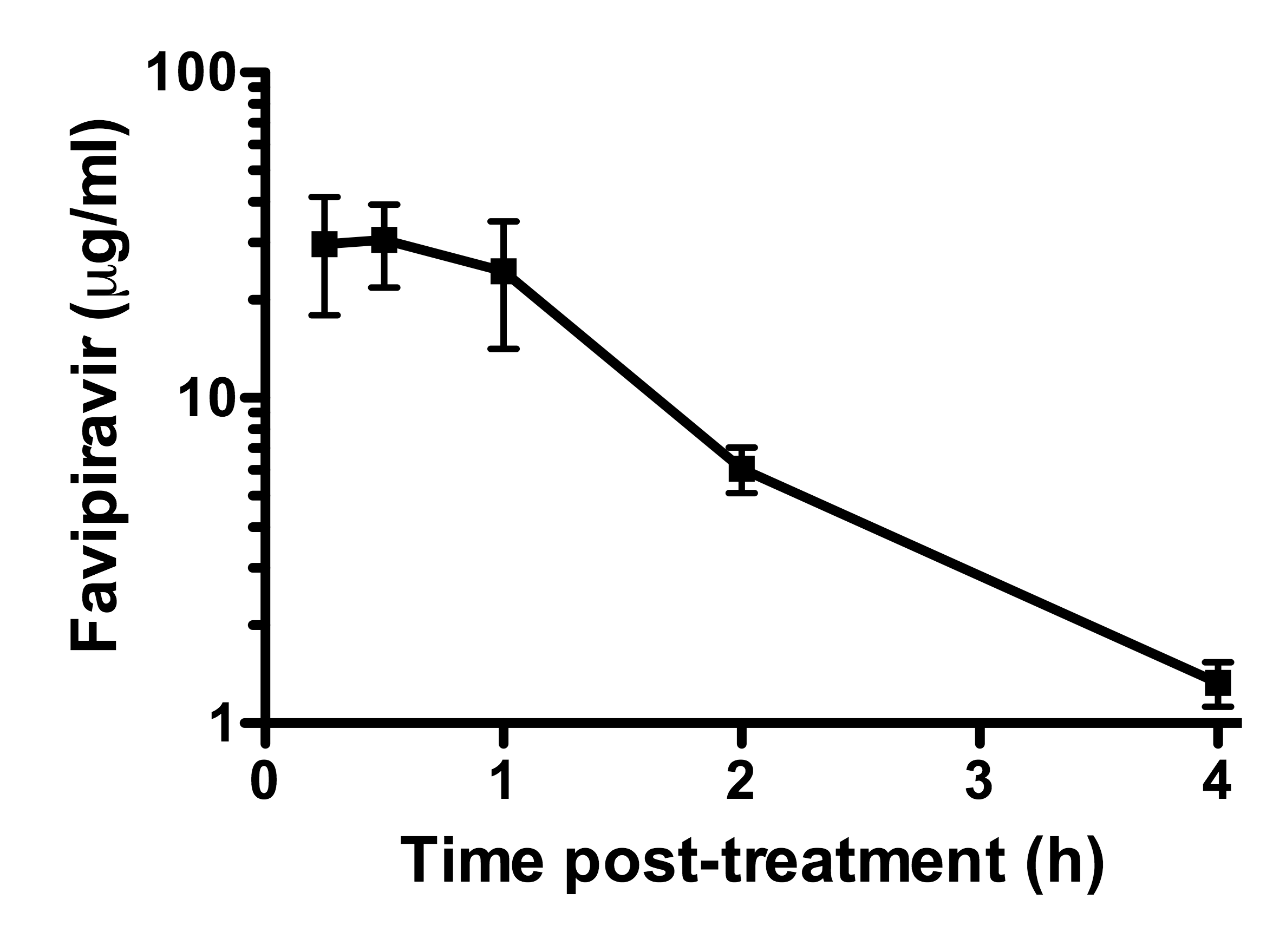

Supplement: Figure S1 — PK analysis of favipiravir in guinea pigs dosed by oral cavity placement. Favipiravir (50 mg/kg) was prepared in carrot food vehicle and administered by placement of the dose towards the back of the palate with a tuberculin syringe. Plasma was collected at 0.25, 0.5, 1, 2 or 4 h after treatment from 3 guinea pigs treated on 2 separate days. Samples were processed and analyzed by HPLC for separation and measurement of favipiravir as described in the methods section. (TIFF) [file pntd.0001342.s001.tiff]
