# Supplementary material for: Effective Oral Favipiravir (T-705) Therapy Initiated after the Onset of Clinical Disease in a Model of Arenavirus Hemorrhagic Fever
Source: PLoS Negl Trop Dis. 2011 Oct 11;5(10):e1342. doi: 10.1371/journal.pntd.0001342 (PMC3191123; doi:10.1371/journal.pntd.0001342)
Supplement: Table S1 — Hematological values during the course of PICV infection in guinea pigsa. a Groups of guinea pigs (n = 3) were sacrificed daily through the course of PICV infection and whole blood was collected for hematologic analysis. b The day-10 group (n = 4) included a moribund guinea pig from the day-11 group. c The day-11 group consisted of 2 guinea pigs. d Total WBC and Gra were significantly increased on day 7 compared to day 1 (P<0.05). e PLT and PCT were significantly lower on days 5–10 and 6–11, respectively, compared to day 1 (P<0.05). f PDW could not be accurately calculated due to depleted platelet counts on days 8–11. WBC, white blood cells; Lym, lymphocytes; Mon, monocytes; Gra, granulocytes; RBC, red blood cells; MCV, mean corpuscular volume; HCT, hematocrit; MCH, mean corpuscular hemoglobin; MCHC, mean corpuscular hemoglogin concentration; RDW, red cell distribution width; Hb, hemoglobin; PLT, platelets; MPV, mean platelet volume; PCT, plateletcrit; and PDW, platelet distribution width. (DOC) [file pntd.0001342.s002.doc]

| **Blood component**  **(units)** | **Day post-infection** | | | | | | | | | |
| --- | --- | --- | --- | --- | --- | --- | --- | --- | --- | --- |
|  | **1** | **3** | **4** | **5** | **6** | **7** | **8** | **9** | **10^b^** | **11^c^** |
| WBC (m/mm^3^) | 2.2 ± 0.5 | 2.8 ± 0.9 | 4.7 ± 0.7 | 3.6 ± 0.7 | 3.8 ± 1.2 | 7.7 ± 1.4^d^ | 4.3 ± 1.0 | 3.2 ± 0.9 | 3.3 ± 1.0 | 3.5 ± 0.1 |
| Lym (m/mm^3^) | 0.8 ± 0.3 | 1.0 ± 0.5 | 1.4 ± 0.1 | 1.2 ± 0.5 | 1.2 ± 0.4 | 1.5 ± 0.3 | 0.9 ± 0.2 | 1.0 ± 0.3 | 0.9 ± 0.3 | 1.0 ± 0.4 |
| Mon (m/mm^3^) | 0.1 ± 0.0 | 0.1 ± 0.1 | 0.2 ± 0.0 | 0.1 ± 0.1 | 0.2 ± 0.1 | 0.2 ± 0.1 | 0.2 ± 0.1 | 0.2 ± 0.1 | 0.2 ± 0.1 | 0.2 ± 0.0 |
| Gra (m/mm^3^) | 1.3 ± 0.3 | 1.7 ± 0.6 | 3.1 ± 0.7 | 2.3 ± 0.5 | 2.5 ± 0.9 | 6.0 ± 1.5^d^ | 3.3 ± 1.0 | 2.1 ± 0.7 | 2.3 ± 0.7 | 2.4 ± 0.2 |
| RBC (m/mm^3^) | 6.0 ± 0.2 | 5.5 ± 0.5 | 5.7 ± 0.2 | 5.3 ± 0.2 | 5.0 ± 0.4 | 5.4 ± 0.4 | 5.5 ± 0.2 | 5.5 ± 0.2 | 5.0 ± 0.5 | 5.1 ± 0.8 |
| MCV (fL) | 84.4 ± 2.6 | 83.9 ± 0.6 | 84 ± 2.7 | 80.1 ± 3.4 | 80.3 ± 1.3 | 81.7 ± 2.4 | 86.0 ± 1.3 | 80.6 ± 3.4 | 80.9 ± 2.7 | 78.1 ± 4.3 |
| HCT (%) | 50.1 ± 0.5 | 46.4 ± 3.8 | 47.5 ± 2.3 | 42.3 ± 1.1 | 40.5 ± 2.7 | 44.3 ± 2.4 | 47.3 ± 2.5 | 44.4 ± 3.2 | 40.6 ± 3.1 | 40.3 ± 8.3 |
| MCH (pg) | 25.1 ± 1.1 | 25.9 ± 1.8 | 25.2 ± 0.9 | 26.2 ± 0.4 | 26.7 ± 0.8 | 26.6 ± 0.8 | 27.2 ± 2.1 | 26.6 ± 0.8 | 26.7 ± 1.7 | 26.1 ± 0.6 |
| MCHC (g/dl) | 29.7 ± 0.5 | 30.9 ± 2.3 | 30.1 ± 0.4 | 32.8 ± 1.9 | 33.2 ± 0.5 | 32.5 ± 0.8 | 31.8 ± 2.5 | 33.0 ± 1.1 | 33.1 ± 1.6 | 33.5 ± 2.7 |
| RDW | 9.3 ± 0.5 | 10.0 ± 1.1 | 9.7 ± 0.7 | 10.1 ± 0.6 | 9.4 ± 0.8 | 9.2 ± 0.3 | 8.8 ± 0.5 | 9.3 ± 0.2 | 9.2 ± 0.6 | 8.6 ± 0.4 |
| Hb (g/dl) | 14.9 ± 0.2 | 14.3 ± 0.8 | 14.3 ± 0.6 | 13.9 ± 0.8 | 13.5 ± 1.0 | 14.4 ± 0.6 | 15.0 ± 1.6 | 14.7 ± 0.7 | 13.4 ± 1.1 | 13.4 ± 1.7 |
| PLT (m/mm^3^) | 173 ± 16 | 158 ± 50 | 137 ± 17 | 89 ± 25^e^ | 51 ± 12^e^ | 70 ± 9^e^ | 24 ± 6^e^ | 37 ± 8^e^ | 23 ± 10^e^ | 27 ± 32 |
| MPV (fL) | 7.3 ± 0.3 | 7.0 ± 0.5 | 7.4 ± 0.4 | 7.3 ± 0.3 | 7.1 ± 0.1 | 7.9 ± 0.6 | 7.9 ± 0.6 | 7.3 ± 0.5 | 7.3 ± 0.5 | 8.2 ± 0.5 |
| PCT (%) | 0.13 ± 0.02 | 0.11 ± 0.04 | 0.10 ± 0.02 | 0.07 ± 0.02 | 0.04 ± 0.01^e^ | 0.05 ± 0.01^e^ | 0.02 ± 0.01^e^ | 0.03 ± 0.01^e^ | 0.02 ± 0.0^e^ | 0.02 ± 0.03^e^ |
| PDW | 8.3 ± 1.1 | 9.0 ± 0.9 | 7.8 ± 2.3 | 6.8 ± 5.9 | 6.9 ± 6.0 | 6.7 ± 5.9 | -^f^ | -^f^ | -^f^ | -^f^ |

^a^ Groups of guinea pigs (n=3) were sacrificed daily through the course of PICV infection and whole blood was collected for hematologic analysis.

^b^ The day-10 group (n=4) included a moribund guinea pig from the day-11 group.

^c^ The day-11 group consisted of 2 guinea pigs.

^d^ Total WBC and Gra were significantly increased on day 7 compared to day 1 (*P* < 0.05).

^e^ PLT and PCT were significantly lower on days 5-10 and 6-11, respectively, compared to day 1 (*P* < 0.05).

^f^ PDW could not be accurately calculated due to depleted platelet counts on days 8-11.

WBC, white blood cells; Lym, lymphocytes; Mon, monocytes; Gra, granulocytes; RBC, red blood cells; MCV, mean corpuscular volume; HCT, hematocrit; MCH, mean corpuscular hemoglobin; MCHC, mean corpuscular hemoglogin concentration; RDW, red cell distribution width; Hb, hemoglobin; PLT, platelets; MPV, mean platelet volume; PCT, plateletcrit; and PDW, platelet distribution width.
