# Supplementary material for: Effective Oral Favipiravir (T-705) Therapy Initiated after the Onset of Clinical Disease in a Model of Arenavirus Hemorrhagic Fever
Source: PLoS Negl Trop Dis. 2011 Oct 11;5(10):e1342. doi: 10.1371/journal.pntd.0001342 (PMC3191123; doi:10.1371/journal.pntd.0001342)
Supplement: Table S2 — Blood chemistry profile during PICV infection in guinea pigsa. a Groups of guinea pigs (n = 3) were sacrificed daily through the course of PICV infection and whole blood was collected for blood chemistry analysis. b The day-10 group (n = 4) included a moribund guinea pig from the day-11 group. c The day-11 group consisted of 2 guinea pigs. d Serum AST was significantly higher on day 10 compared to day 1 (P<0.05). e Serum ALB was significantly lower on days 9 and 10 compared to day 1 (P<0.05). f Values were below the limit of detection (50 mg/dL) in all three guinea pigs. AST, aspartate aminotransferase; AMY, amylase; ALP, alkaline phosphatase; ALT, alanine aminotransferase; BUN, blood urea nitrogen; ALB, albumin; TBIL, total bilirubin; Ca, calcium; CRE, creatinine; GGT, gamma-glutamyl transferase; GLU, glucose; TP, total protein; TBIL, total bilirubin;. ALB, Albumin; CHO, cholesterol; IP, inorganic phosphate. (DOC) [file pntd.0001342.s003.doc]

| **Blood component**  **(units)** | **Day p.i.** | | | | | | | | | |
| --- | --- | --- | --- | --- | --- | --- | --- | --- | --- | --- |
|  | **1** | **3** | **4** | **5** | **6** | **7** | **8** | **9** | **10^b^** | **11^c^** |
| AST (U/L) | 40 ± 3 | 35 ± 4 | 33 ± 5 | 43 ± 6 | 57 ± 43 | 67 ± 8 | 221 ± 120 | 231 ± 107 | 350 ± 159^d^ | 249 ± 11 |
| AMY (U/L) | 2186 ± 40 | 1638 ± 338 | 1620 ±31 | 1589 ± 121 | 1675 ± 267 | 1414 ± 171 | 1539 ± 157 | 1679 ± 163 | 1856 ± 544 | 1869 ± 390 |
| ALP (U/L) | 303 ± 25 | 260 ± 26 | 257 ± 14 | 265 ± 37 | 229 ± 52 | 288 ± 100 | 414 ± 66 | 401 ± 140 | 505 ± 152 | 355 ± 218 |
| ALT (U/L) | 26 ± 4 | 25 ± 1 | 27 ± 2 | 32 ± 3 | 25 ± 8 | 27 ± 3 | 29 ± 8 | 30 ± 6 | 39 ± 24 | 26 ± 4 |
| BUN (mg/dL) | 17.4 ± 1.1 | 11.9 ± 0.6 | 13.0 ± 1.4 | 12.0 ± 1.5 | 12.3 ± 0.7 | 12.4 ± 0.4 | 14.0 ± 2.5 | 16.3 ± 1.2 | 20.5 ± 7.7 | 15.8 ± 2.4 |
| Ca (mg/dL) | 12.7 ± 0.5 | 12.6 ± 0.5 | 12.8 ± 0.5 | 12.1 ± 0.2 | 12.9 ± 0.5 | 12.9 ± 0.5 | 12.4 ± 0.5 | 12.2 ± 0.7 | 11.9 ± 0.2 | 11.6 ± 1.3 |
| CRE (mg/dL) | 0.3 ± 0.1 | 0.3 ± 0.1 | 0.4 ± 0.1 | 0.3 ± 0.0 | 0.3 ± 0.0 | 0.4 ± 0.0 | 0.3 ± 0.1 | 0.4 ± 0.1 | 0.4 ± 0.1 | 0.4 ± 0.0 |
| GGT (U/L) | 13 ± 2 | 16 ± 3 | 15 ± 3 | 14 ± 2 | 16 ± 4 | 16 ± 2 | 16 ± 3 | 18 ± 3 | 16 ± 3 | 18 ± 3 |
| GLU (mg/dL) | 210 ± 40 | 215 ± 10 | 180 ± 40 | 196 ± 21 | 226 ± 23 | 218 ± 19 | 170 ± 6 | 181 ± 20 | 192 ± 46 | 181 ± 18 |
| TP (g/dl) | 4.8 ± 0.0 | 4.4 ± 0.5 | 4.4 ± 0.4 | 4.2 ± 0.2 | 4.2 ± 0.3 | 4.5 ± 0.3 | 4.4 ± 0.4 | 4.5 ± 0.2 | 4.1 ± .0.1 | 3.9 ± 0.3 |
| TBIL (mg/dL) | 0.1 ± 0.0 | 0.1 ± 0.0 | 0.2 ± 0.1 | 0.1 ± 0.0 | 0.1 ± 0.0 | 0.1 ± 0.0 | 0.1 ± 0.0 | 0.1 ± 0.0 | 0.2 ± 0.1 | 0.3 ± 0.1 |
| ALB (g/dL) | 2.8 ± 0.1 | 2.5 ± 0.2 | 2.4 ± 0.2 | 2.2 ± 0.1 | 2.2 ± 0.1 | 2.4 ± 0.1 | 2.4 ± 0.3 | 2.0 ± 0.6^e^ | 2.1 ± 0.1^e^ | 2.1 ± 0.1 |
| CHO (mg/dL) | 51 ± 1 | <50^f^ | <50^f^ | <50^f^ | <50^f^ | 55 ± 9 | 67 ± 9 | 64 ± 20 | 54 ± 9 | 54 ± 3 |
| IP (mg/dL) | 9.8 ± 0.9 | 10.0 ± 0.1 | 9.4 ± 0.4 | 8.5 ± 0.3 | 8.7 ± 0.2 | 9.7 ± 0.9 | 10.2 ± 0.7 | 9.3 ± 0.4 | 9.5 ± 0.7 | 7.5 ± 0.3 |

^a^ Groups of guinea pigs (n=3) were sacrificed daily through the course of PICV infection and whole blood was collected for blood chemistry analysis.

^b^ The day-10 group (n=4) included a moribund guinea pig from the day-11 group.

^c^ The day-11 group consisted of 2 guinea pigs.

^d^ Serum AST was significantly higher on day 10 compared to day 1 (*P* < 0.05).

^e^ Serum ALB was significantly lower on days 9 and 10 compared to day 1 (*P* < 0.05).

^f^ Values were below the limit of detection (50 mg/dL) in all three guinea pigs.

AST, aspartate aminotransferase; AMY, amylase; ALP, alkaline phosphatase; ALT, alanine aminotransferase; BUN, blood urea nitrogen; ALB, albumin; TBIL, total bilirubin; Ca, calcium; CRE, creatinine; GGT, gamma-glutamyl transferase; GLU, glucose; TP, total protein; TBIL, total bilirubin;. ALB, Albumin; CHO, cholesterol; IP, inorganic phosphate.
