# Supplementary material for: Effective Oral Favipiravir (T-705) Therapy Initiated after the Onset of Clinical Disease in a Model of Arenavirus Hemorrhagic Fever
Source: PLoS Negl Trop Dis. 2011 Oct 11;5(10):e1342. doi: 10.1371/journal.pntd.0001342 (PMC3191123; doi:10.1371/journal.pntd.0001342)
Supplement: Table S3 — Hematology and blood chemistry analysis of guinea pigs treated for 10 days with favipiravira. a Guinea pigs (n = 5/group) treated orally twice daily for ten days with the indicated doses of favipiravir and sacrificed 7 days after the final dose was administered. Whole blood and sera were analyzed for hematology and blood chemistry. WBC, white blood cells; Neu, neutrophils; Lym, lymphocytes; Mon, monocytes; Eos, eosinophils; Bas, basophils; RBC, red blood cells; Hb, hemoglobin; HCT, hematocrit; MCV, mean corpuscular volume; MCH, mean corpuscular hemoglobin; MCHC, mean corpuscular hemoglogin concentration; RDW, red cell distribution width; PLT, platelets; MPV, mean platelet volume; ALB, albumin; ALP, alkaline phosphatase; ALT, alanine aminotransferase; AMY, amylase; TBIL, total bilirubin; BUN, blood urea nitrogen; Ca, calcium, PHOS, phosphate; CRE, creatinine; GLU, glucose; Na+, sodium; K+, potassium; TP, total protein; GLOB, globulin. (DOC) [file pntd.0001342.s004.doc]

**Blood**

**Component 500 250 100**

**(units) mg/kg/d mg/kg/d mg/kg/d Placebo**

**HEMATOLOGY**

WBC (K/μL) 3.4 ± 1.2 3.6 ± 1.2 3.8 ± 0.4 3.3 ± 1.7

Neu (K/μL) 1.5 ± 0.6 1.4 ± 0.5 1.5 ± 0.3 1.4 ± 0.6

Neu (%) 44.2 ± 5.8 40.2 ± 7.2 40.4 ± 5.5 43.4 ± 8.5

Lym (K/μL) 1.7 ± 0.7 2.1 ± 0.8 2.2 ± 0.3 1.9 ± 1.1

Lym (%) 52.6 ± 6.3 57.7 ± 6.8 57.7 ± 4.8 54.9 ± 8.5

Mon (K/μL) 0.1 ± 0.0 0.1 ± 0.0 0.1 ± 0.0 0.1 ± 0.1

Mon (%) 1.9 ± 0.5 1.6 ± 0.7 1.6 ± 1.1 1.7 ± 0.6

Eos (K/μL) 0.0 ± 0.0 0.0 ± 0.0 0.0 ± 0.0 0.0 ± 0.0

Eos (%) 1.0 ± 1.1 0.3 ± 0.3 0.2 ± 0.2 0.1 ± 0.1

Bas (K/μL) 0.0 ± 0.0 0.0 ± 0.0 0.0 ± 0.0 0.0 ± 0.0

Bas (%) 0.2 ± 0.3 0.1 ± 0.1 0.1 ± 0.1 0.0 ± 0.0

RBC (M/μL) 5.9 ± 0.4 6.2 ± 0.7 5.6 ± 0.2 5.8 ± 0.4

Hb (g/dL) 14.3 ± 0.6 14.7 ± 1.6 13.5 ± 0.6 5.8 ± 0.4

HCT (%) 47.9 ± 2.5 48.8 ± 5.4 45.4 ± 2.6 59.1 ± 18.4

MCV (fL) 81.3 ± 2.6 79.3 ± 2.5 80.4 ± 2.0 81.2 ± 2.0

MCH (pg) 24.2 ± 1.0 23.9 ± 1.3 23.9 ± 0.2 24 ± 0.8

MCHC (g/dL) 24.2 ± 1.0 23.9 ± 1.3 23.9 ± 0.2 24 ± 0.8

RDW (%) 15.4 ± 1.3 14.9 ± 1.1 15.4 ± 0.6 14.7 ± 0.8

PLT (K/μL) 432.0 ± 61.0 360.0 ± 25.0 389.0 ± 57.0 427.0 ± 61.0

MPV (fL) 5.4 ± 0.3 5.4 ± 0.5 5.4 ± 0.4 5.7 ± 0.5

**BLOOD CHEMISTRY**

ALB (g/dL) 3.7 ± 0.3 3.6 ± 0.2 3.6 ± 0.2 3.7 ± 0.1

ALP (U/L) 286 ± 42 299 ± 33 340 ± 27 302± 44

ALT (U/L) 30 ± 5 36 ± 11 35 ± 3 34 ± 5

AMY (U/L) 1265 ± 185 1195 ± 125 1135 ± 158 1072 ± 62

TBIL (mg/dL) 0.2 ± 0.0 0.2 ± 0.0 0.2 ± 0.0 0.2 ± 0.0

BUN (mg/dL) 13.2 ± 1.8 13.8 ± 2.2 12.6 ± 0.5 12.8± 0.4

Ca (mg/dL) 12.6 ± 0.7 12.2 ± 0.5 12.6 ± 0.5 13.0 ± 0.3

PHOS (mg/dL) 9.7 ± 1.1 9.6 ± 0.6 9.5 ± 0.6 10.8 ± 0.6

CRE (mg/dL) 0.3 ± 0.1 0.4 ± 0.0 0.4 ± 0.1 0.4 ± 0.1

GLU (mg/dL) 175 ± 27 159 ± 26 165 ± 35 187 ± 39

Na^+^ (mM) 8.5 ± 0.0 8.4 ± 0.1 8.5 ± 0.0 8.5 ± 0.0

K^+^ (mM) 8.5 ± 0.0 8.4 ± 0.1 8.5 ± 0.0 8.5 ± 0.0

TP (g/dL) 4.7 ± 0.4 4.5 ± 0.3 4.4 ± 0.2 4.6 ± 0.2

GLOB (g/dL) 1.0 ± 0.2 0.8 ± 0.2 0.9 ± 0.2 0.9± 0.2

^a^ Guinea pigs (n=5/group) treated orally twice daily for ten days with the indicated doses of favipiravir and sacrificed 7 days after the final dose was administered. Whole blood and sera were analyzed for hematology and blood chemistry

WBC, white blood cells; Neu, neutrophils; Lym, lymphocytes; Mon, monocytes; Eos, eosinophils; Bas, basophils; RBC, red blood cells; Hb, hemoglobin; HCT, hematocrit; MCV, mean corpuscular volume; MCH, mean corpuscular hemoglobin; MCHC, mean corpuscular hemoglogin concentration; RDW, red cell distribution width; PLT, platelets; MPV, mean platelet volume; ALB, albumin; ALP, alkaline phosphatase; ALT, alanine aminotransferase; AMY, amylase; TBIL, total bilirubin; BUN, blood urea nitrogen; Ca, calcium, PHOS, phosphate; CRE, creatinine; GLU, glucose; Na^+^, sodium; K^+^, potassium; TP, total protein; GLOB, globulin.
